# Multi-modal virtual reality system for tinnitus treatment methods and validation

**DOI:** 10.1371/journal.pone.0330843

**Published:** 2025-09-08

**Authors:** Hojun Aan, Kang Hyeon Lim, Jihoo Kim, Hong Jin Kim, Ye Hwan Lee, Eunjin Kim, Yoon Chan Rah, Intae Hwang, Euijin Kim, Sungkean Kim, Jimmon Kim, Jiake Yang, Sangsun Han, Kibum Kim, June Choi

**Affiliations:** 1 Department of Human-Computer Interaction, Hanyang University, Ansan, Republic of Korea; 2 Department of Otorhinolaryngology-Head and Neck Surgery, Ansan Hospital, Korea University College of Medicine, Ansan, Republic of Korea; 3 Department of Interdisciplinary Robot Engineering Systems, Hanyang University, Ansan, Republic of Korea; 4 Healthcare Readiness Institute for Unified Korea, Korea University College of Medicine, Seoul, Republic of Korea; 5 Bionics Research Center, Biomedical Research Division, Korea Institute of Science and Technology (KIST), Seoul, Republic of Korea; 6 Department of Medical Informatics, Korea University College of Medicine, Seoul, Republic of Korea; Brown University Warren Alpert Medical School, UNITED STATES OF AMERICA

## Abstract

Virtual reality (VR) has been utilized in clinical treatment because it can efficiently simulate situations that are difficult to control in the real world. In this study, we evaluated the efficacy of VR in patients with chronic subjective tinnitus. We assessed the clinical effectiveness based on electroencephalogram (EEG) analysis and questionnaire responses after patients participated in a 6–8-week VR-based tinnitus relief program. The intervention involved removing tinnitus avatars in the VR, through which we expected the patients to experience subjective tinnitus control. Standardized low-resolution brain electromagnetic tomography was used to analyze changes in source activity in prefrontal regions associated with tinnitus. The study included patients aged 27–68 years with chronic non-pulsatile tinnitus lasting ≥3 months. Patients completed VR sessions, neurological EEGs, and questionnaires. Statistically significant differences were observed in the tinnitus handicap questionnaire total scores immediately after treatment (p = 0.002) and 1-month post-treatment (p = 0.001) compared to those before treatment. Significant changes were also found in the visual numeric scale and profile of mood states scores 1-month post-treatment. Additionally, significant changes were observed in sensor-level and source-level power spectrum criteria immediately following the VR experiment, suggesting that similar to some forms of cognitive behavioral therapy, VR-based programs may help alleviate tinnitus-related distress in patients with chronic subjective tinnitus.

## Introduction

Tinnitus is the perception of sounds by a person in the absence of external acoustic stimuli. Severe tinnitus can significantly affect daily life and induce anxiety and other negative emotions. Tinnitus is categorized into two main types: objective tinnitus, caused by a physiological process and audible to an external observer, and subjective tinnitus, which is only perceptible to the patient [1,2]. Subjective tinnitus is defined exclusively based on self-reported experiences. The objective of treating subjective tinnitus is not to eliminate tinnitus directly but rather to alleviate the stress it causes [3]. Cognitive behavioral therapy (CBT) is the most recommended and commonly used method among the various treatments for subjective tinnitus. CBT helps patients reframe negative thoughts that cause distress and improves symptoms through relaxation techniques, exposure to stimuli, and behavioral interventions [4].

Virtual reality (VR) can be integrated into CBT for patients with difficulty expressing their tinnitus experience. In traditional CBT, patients typically identify and discuss their experiences of tinnitus. However, in VR therapy, patients only need to express tinnitus in a simple manner without discussing their experience. Furthermore, CBT requires patients to adjust their perspectives towards tinnitus, enabling them to apply counseling insights to their personal lives. The use of VR techniques provides a new approach to tinnitus treatment, allowing patients to experience tinnitus sounds directly, without needing to alter their perception of tinnitus. Researchers have developed a VR treatment for tinnitus that reproduces a patient’s tinnitus and utilizes a head-related transfer function (HRTF) to create a spatial audio representation of tinnitus [5]. In a clinical trial using this system, patients exhibited remission of tinnitus symptoms, with a notable reduction in the Subjective Tinnitus Severity Scale (STSS) score [6]. However, the VR system used in the study only allowed basic interactions by observing and approaching the tinnitus. This limits the ability of the patients to discern auditory variations at different proximities. Additionally, few studies have used VR or augmented reality technology in the field of tinnitus [7,8].

This study presents a VR system developed for the treatment of tinnitus that incorporates haptic feedback to provide participants with multi--modal sensations. A VR eye-tracking and haptic system was devised to enable patients to interact with a “tinnitus avatar,” which represents their tinnitus symptoms in a physical form. By visualizing tinnitus as an interactive object in a VR environment, patients can eliminate tinnitus via touch. We correlated haptic feedback with audiovisual feedback during tinnitus erasure. Our system produces a more immersive experience by reducing the sound and haptic feedback as the tinnitus avatar shrinks.

To verify the effectiveness of the system, we conducted a clinical trial on the participants with tinnitus. We used the tinnitus handicap inventory (THQ) questionnaire to evaluate subjective tinnitus along with various quality of life and VR questionnaires for self-reporting. Electroencephalography (EEG) was used to verify the objective effects of the treatment. This study aimed to demonstrate the feasibility of our novel VR treatment for tinnitus.

## Methods

### Participants and questionnaires

**Fig 1:**
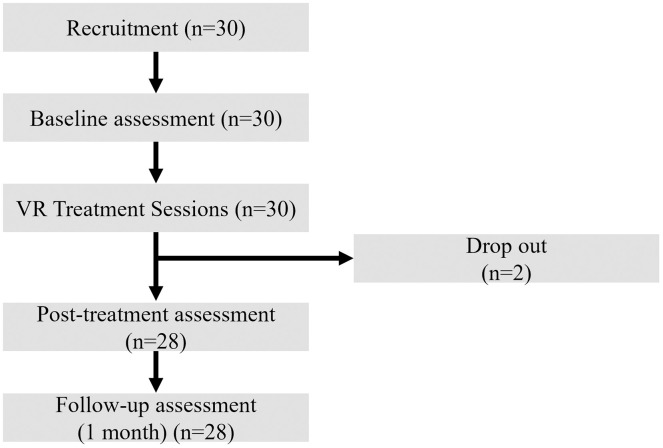
Participant flow diagram.

This study included patients aged 27–68 years who visited the Department of Otorhinolaryngology at Korea University Ansan Hospital with tinnitus as their chief complaint and had chronic non-pulsatile tinnitus persisting for at least 3 months. The participants were required to be fluent in Korean and consent to participate. Patients with otitis media, Meniere’s disease, or inner ear disorders; those frequently exposed to loud noise due to occupation or hobbies; and those expected to have difficulty operating a head-mounted display (HMD) for VR programs or face challenges in completing the given questionnaires without errors were excluded from the study. Ultimately, 28 patients with tinnitus (15 males and 13 females) participated in the study. The flow of participants through the enrollment, intervention, and analysis stages of the study is detailed in Fig 1. Power analysis using G*Power 3.1.9.7 version [9] indicated that a minimum of 18 participants was required for statistical power, assuming an effect size (f) of 0.4, an alpha level of 0.05, and a power of 0.95. The sample size of 28 patients was selected based on the effect size and variability observed in similar studies, like the one that used 19 patients [7].

Medical records, demographic information, physical examinations, and otological examinations were evaluated. All the participants underwent pre- and post-experimental hearing and tinnitus assessments. Hearing thresholds were measured at seven octave frequencies (0.125, 0.25, 0.5, 1, 2, 4, and 8 kHz) using pure tone audiometry. For tinnitus pitch matching, a two-alternative forced-choice procedure was applied with auditory stimuli presented at 30 dB SL between 0.125 and 16 kHz in the contralateral ear. Subsequently, the tinnitus loudness level was adjusted in 5 dB steps according to the ipsilateral hearing threshold at that frequency.

The pre- and post-experimental statuses of the participants regarding tinnitus were assessed through questionnaires, including the THQ, and a VNS related to the severity of tinnitus-related distress. Additionally, questionnaires addressing symptoms associated with tinnitus, such as the PSQI, WHO-QoL, POMS, and HADS for evaluating depression, anxiety, and sleep disorders accompanying tinnitus, were completed.

### Experimental design

We employed a within-participants experimental design. The EEG and questionnaire scores after completing each treatment session were compared before and after the full treatment program.

Participants visited the clinic three times, once every 2 weeks, for this experiment. During the initial visit, experimenters obtained signed consent from the participants. Participants underwent endoscopic tympanometry and hearing/tinnitus testing. During the first visit, we performed an EEG and administered a questionnaire, and the participants underwent the first treatment in a virtual distance environment. During the next visit, participants used the dining room and living room environments for training in the second and third treatments, respectively. In the last visit, they used the bedroom environment, followed by another EEG and questionnaire.

Subsequently, artifacts were removed via independent component analysis (ICA) and EEG researcher-given epochs where the amplitude of each electrode exceeded ± 75 uV. Finally, a set of 30 flawless epochs was constructed for each participant. The aforementioned preprocessing method is one of the most commonly used methods for EEG preprocessing.

### Environment protocol

**Fig 2:**
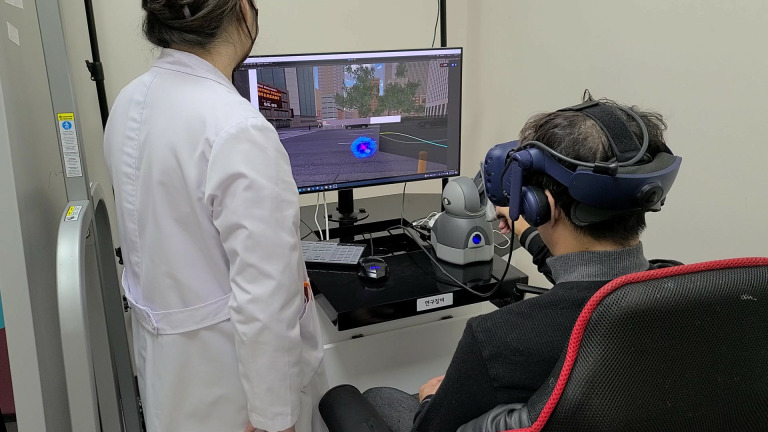
A patient engaged in VR gameplay. The visualization of a virtual tinnitus object is represented by a blue sphere-shaped virtual image. VR, virtual reality.

The VR system software was developed using the Unity 2021.1.6f1 game development engine, and the hardware used was a Vive Eye VR Pro HMD. During the VR treatment, the participants interacted with a virtual tinnitus avatar using a pen-shaped haptic device from Open Haptics Touch (3D Systems Corporation, SC, USA). The experimental setup is detailed in Fig 2. The black box on the right side of the monitor displays a waveform reflecting the current tinnitus state, whereas the white bar of the tinnitus avatar remains visible throughout the simulation. The patient interacted with the tinnitus avatar using the Open haptics pen.

The HRTF integrates spatial acoustics into virtual environments. This is achieved by calculating and adjusting the time for the sound to reach each ear and the pitch of the sound individually for each ear in response to head movements. HRTF uses acoustic parallax, which is the relative angular gap between the sound source position in the virtual environment and the two ears of the user, to generate a spatial sound effect [10]. Stereo panning is an alternative method for obtaining spatial sounds that modulate the volume of each ear differently. However, stereo panning does not provide the same level of acoustic parallax. A comparative study has demonstrated that HRTF allows users to perceive the direction of sounds more naturally than stereo, creating a more immersive treatment experience [11]. Consequently, this study utilized Resonance Audio, a software development kit developed by Google that integrates various HRTFs. In our study, the spatial acoustic effect was applied to both the virtual tinnitus and ambient sounds.

Previous studies have demonstrated the benefits of spatially controlled multi-modal virtual perception for the treatment of tinnitus [6]. Therefore, our VR system provided patients with four virtual environments: bedroom, living room, dining room, and street.

**Fig 3:**
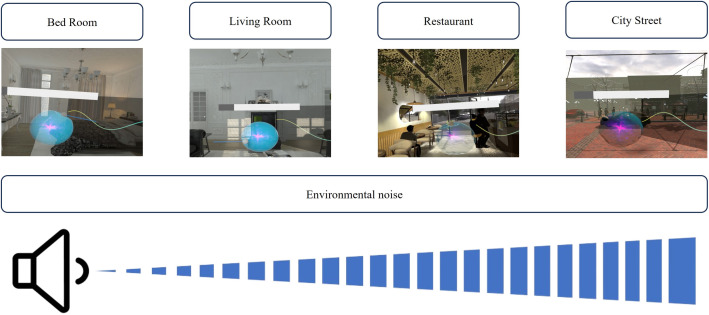
Examples of virtual reality tinnitus treatment scenes. The scenes were arranged from left to right based on increasing environmental noise. Participants experienced the scenes sequentially from left to right.

Each environment incorporated two audible voices: a virtual tinnitus avatar that produced tinnitus sounds and environmental sounds. For instance, the dining room environment includes the sounds of people talking, whereas the living room includes the sound of a TV. The environmental sounds were spatialized and heard simultaneously with the tinnitus sounds. This sound design allows patients to control tinnitus in an environment that closely resembles the real world. The virtual environment was presented in a logical order, with ambient noise progressing from loud to quiet, starting with the street, followed by the dining room, living room, and bedroom. The level of noise in each room is shown in Fig 3. This space technique allowed the patients to gradually experience the increasing sound of their tinnitus and learn how to control it in different environments. Consequently, the patient could perceive tinnitus fading away as it became buried in everyday sounds.

**Fig 4:**
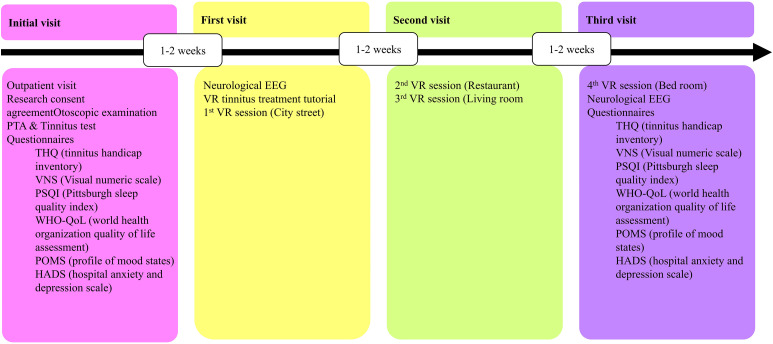
Workflows and tasks for the overall progression of the experiment. VR, virtual reality; EEG, electroencephalography; PTA, pure tone audiometry; THQ, tinnitus handicap questionnaire; WHO-QoL, World Health Organization quality of life assessment; POMS, profile of mood states; HADS, hospital anxiety and depression scale; PSQI, Pittsburgh sleep quality index; VNS, visual numeric scale.

The virtual environment presented by the system to the patient is shown in Fig 4. The system operates as follows. First, the tinnitus sound is customized for each patient. Based on the patients’ description of their tinnitus and tinnitus test results, tinnitus sounds are synthesized using Sample VAE [12] to provide an in-game tinnitus sound as similar as possible to their tinnitus sound. In the virtual environment, a virtual tinnitus avatar that emits synthesized sounds is introduced. At the initiation of the treatment, the patient actively tracks the virtual tinnitus sound, guided by spatial acoustics, to locate the virtual tinnitus avatar. Upon successfully locating the avatar, the patient is instructed to maintain visual focus on the avatar for a duration of 3 s. When the patient focuses their gaze on the tinnitus avatar, a red circle appears in front of the avatar, indicating that the participants are appropriately focusing their eyes on it.

The tinnitus avatar is brought directly in front of the patient after successfully focusing their gaze for 3 s. Subsequently, the patient is presented with a black screen featuring a waveform graph with a white bar positioned behind it. The white bar decreases as the patient interacts with the tinnitus avatar; when it reaches the end, the tinnitus avatar disappears. The waveform graph depicts the shape of the tinnitus waveform, with the peak weakening as the avatar’s health decreases, providing a visual cue indicating the gradual disappearance of tinnitus.

With the avatar positioned in front of the patient, manipulation of the Open Haptics pen is employed to press, rub, and remove the tinnitus avatar. The patients receive haptic feedback throughout the process. As the patient rubs the avatar and the tinnitus avatar’s health decreases, the tinnitus avatar bubbles crumble, and the sound becomes quieter, providing a visual and auditory signal that the tinnitus is fading away. When the tinnitus avatar’s health value becomes zero, it disappears. This process was repeated thrice over the course of treatment, and the initial location of the virtual tinnitus avatar differed at each treatment time.

A clinical trial involving participants diagnosed with tinnitus was conducted to assess the efficacy of this system. The workflow and tasks for the experiment are shown in Fig 4. We used the THQ questionnaire, which assesses the symptoms of tinnitus, a questionnaire about the activities of daily life that can be affected by tinnitus, and a questionnaire about the severity of motion sickness in VR as validation tools. For the quantitative evaluation, an EEG was used as a testing tool.

### Data acquisition and analysis

A QEEG-32FX amplifier (LAXTHA Inc., Korea) was used to record the EEG signals. It features 32 Ag-AgCl channels affixed to a cap with an extended 10–20 positioning scheme. The ground channel was attached to the left mastoid, while the reference channel was placed on the right mastoid. EEG data were acquired using a band-pass filter while the participant was in a resting state, with eyes closed. The cutoff frequency of the filter ranged from 0 to 1000 Hz, and the data sampling rate was set to 250 Hz.

The acquired EEG data were preprocessed using the EEGLAB v14.1.2 toolbox and MATLAB R2021b (MathWorks, Natick, MA, USA). Gross artifacts were deemed unacceptable upon visual examination by a knowledgeable individual with no prior information regarding the source of the data. Artifacts associated with eye movements or blinks were rectified by implementing an ICA procedure in the preprocessing software. To filter the data, we applied a 1–55 Hz band-pass filter. Following this procedure, the signal underwent common average referencing, which was then succeeded by baseline correction via the elimination of the DC offset for every channel. Finally, the signal was divided into distinct 2-s epochs, with all overlaps between them meticulously avoided. All epochs containing substantial physiological anomalies (amplitude surpassing ±100 μV) at any of the 32 electrodes were discarded. A random selection of 30 artifact-free epochs was extracted for each participant from the remaining epochs, considering temporal bias. The number of epochs was ascertained based on the varying number of residual epochs exhibited by each participant after the rejection of artifacts. Furthermore, an earlier investigation established acceptable reliability of resting-state EEG data exceeding 40 s.

### Source localization using sLORETA

In this study, we employed the standardized low-resolution brain electromagnetic tomography (sLORETA) software, a widely acknowledged method in EEG source imaging, to analyze scalp-recorded EEG data for intracerebral electrical sources. sLORETA effectively addresses the EEG inverse problem by assuming that the neuronal activity of a given voxel is closely related to that of its neighboring voxels [13]. The preprocessed EEG data were analyzed for PSD across six frequency bands: delta (1–4 Hz), theta (4–8 Hz), alpha (8–12 Hz), low beta (12–18 Hz), high beta (18–30 Hz), and gamma (30–55 Hz). In sLORETA, cerebral sources are spatially represented by 6239 voxels, each with a 5-mm resolution, encompassing the amygdala, hippocampus, and cortical gray matter [14]. The localization of these sources was based on the digitized Montreal Neurological Institute 152 brain template and was subsequently transformed into Talairach coordinates for precision [15]. Time series analysis of the cortical sources at each of the 152 coordinates was conducted, and the data was band-pass filtered into six specified frequency bands.

### Statistical analysis

In this investigation, we aimed to quantify the level of alleviation of tinnitus-related distress by examining potential EEG biomarkers from an exploratory perspective. Data analysis was performed using SPSS version 20.0 software (SPSS, Chicago, Illinois, USA). Paired t-tests were conducted to assess differences in PSD across six predefined frequency bands and related source activities. Comparisons were made between two sets of data: one acquired before and after the VR intervention, and the other obtained before the VR intervention and 1-month post-intervention. In light of the exploratory nature of this study, paired t-tests were conducted separately to compare pre- and post-treatment EEG data, as well as pre-treatment and one-month follow-up EEG data. Given the primary focus on detecting intervention effects, corrections for multiple comparisons were not applied, and the significance threshold was set at p < 0.05 (two-tailed).

For the questionnaire, paired t-tests were initially performed in accordance with the EEG analysis. Temporal changes were then examined using repeated measures ANOVA, with post-hoc pairwise comparisons conducted using Bonferroni-adjusted paired t-tests.

### Ethical approval statement

The study received ethical approval from the Institutional Review Board and Ethics Committee of Korea University Ansan Hospital (No. 2021AS0381). The recruitment period for the study began on 21/09/2022 and ended on 04/12/2023. Participants were assured that their participation was entirely voluntary, with the right to withdraw at any time without impacting their medical care at the hospital. The researcher emphasized that all data collected would be used solely for research purposes and stored confidentially in the principal investigator’s office. All individuals who agreed to participate provided written, informed consent.

The trial was registered at https://cris.nih.go.kr under the registry number KCT0007538, with the full date of first registration being 21/09/2022. All experiments were conducted in accordance with the relevant guidelines and regulations.

## Results

### Patient demographics and clinical features

A total of 28 patients with nonpulsatile tinnitus completed all VR sessions, neurological EEG, and questionnaires. The mean age of the participants was 55.93 ± 9.90 years. All participants had experienced tinnitus symptoms for more than 3 months. Overall, 14 patients experienced tinnitus in both ears, 12 in the left ear, and 2 in the right ear. The average loudness of tinnitus in the 28 ears was 6.67 ± 10.33 dB SL, and the average pitch was 5.09 ± 3.09 kHz (Table 1).

**Table 1:** General patient information.

Clinical characteristics (n = 28)	
Age (years)	55.93 ± 9.90^a^
Sex	
Male	15 (53.6%)
Female	13 (46.4%)
Hearing level (dB HL) ^b^	
Right (28 ears)	23.35 ± 12.51^a^
Left (28 ears)	32.50 ± 15.89^a^
Tinnitus laterality (right/left/both)	2/12/14
Tinnitus loudness (dB SL)	6.67 ± 10.33^a^
Right (16 ears)	5.94 ± 7.34^a^
Left (26 ears)	7.12 ± 11.78^a^
Tinnitus pitch matching (kHz)	5.09 ± 3.09^a^
Right (16 ears)	5.45 ± 2.75^a^
Left (26 ears)	4.88 ± 3.27^a^

dB SL, decibel sensation level; kHz, kilohertz.

^a^ Mean ± standard deviation.

^b^ Average of four frequencies (0.5, 1, 2, and 4 kHz).

### Analysis of the questionnaires administered to the study participants

**Fig 5:**
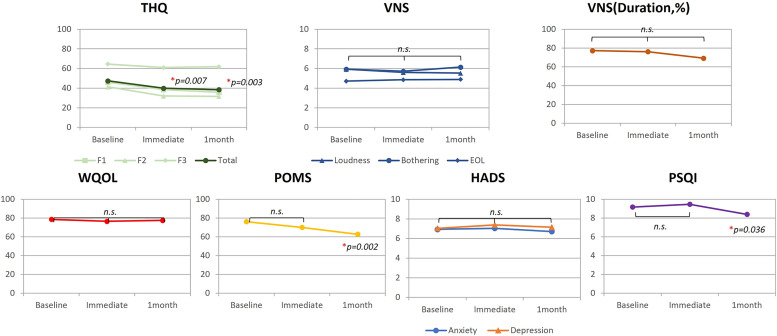
Results of the questionnaires administered to the study participants. THQ, tinnitus handicap questionnaire; LD, loudness score; AN, annoyance score; EOL, effect on life score; WHO-QoL, World Health Organization quality of life assessment; POMS, profile of mood states; HADS, hospital anxiety and depression scale; PSQI, Pittsburgh sleep quality index; VNS, visual numeric scale.

The questionnaires were conducted at three distinct time points: prior to the treatment (Pre), immediately following the treatment (Post), and one month following the treatment (1M). The statistical summaries presented in S1 Table. To evaluate changes in questionnaire scores over time, we conducted repeated measures ANOVAs followed by Bonferroni-adjusted paired t-tests. The repeated measures ANOVA revealed significant main effects of time for the THQ (p < .001), PSQI (p = 0.041), and POMS (p = 0.015). No significant changes were found over time for the WQOL, HADS (both Anxiety and Depression subscales), or most measures of the VNS, including loudness, annoyance, and effect on life (all p > 0.05). The results are shown in Fig 5 and Table 2. The de-identified, individual-level questionnaire response data are provided in S1 Dataset, ensuring that the study’s findings are fully reproducible.

**Table 2:** Repeated measures ANOVA analyses for questionnaires.

Factor	Sum of Squares (SS)	df	Mean Square (MS)	F Value	Partial Eta Squared (η²)	Observed Power (1-β)	p-Value
THQ	1272.725	2	636.363	10.093	0.272	0.981	<.001*
PSQI	17.238	2	8.619	3.387	0.111	0.614	.041*
WQOL	62.167	2	31.083	1.972	0.068	0.391	.149
POMS	2546.167	2	1273.083	4.565	0.145	0.752	.015*
HADS -Anxiety	1.500	2	0.750	0.380	.014	0.108	.685
HADS -Depression	1.881	2	0.940	0.458	.017	0.121	.635
VNS -Duration	1046.167	2	523.083	2.757	.093	0.522	.072

* *p* indicates statistical significance.

Post-hoc analyses with Bonferroni-adjustment indicated that THQ scores significantly decreased from pre-treatment to post-treatment (p = 0.007) and from pre-treatment to 1-month post-treatment (p = 0.003). For the PSQI, a significant improvement was observed from post-treatment to 1-monthpost-treatment (p = 0.036). Regarding the POMS, scores significantly improved from pre-treatment to 1-month post-treatment (p = 0.002). Detailed results post-hoc tests are presented in Table 3.

**Table 3:** Bonferroni-adjusted post-hoc analyses for questionnaires.

Measure	Comparison	Mean Difference (M)	Standard Error (SE)	95% CI Lower	95% CI Upper	t-statistic	df	p-Value
THQ	Pre vs Post	7.406	2.207	1.773	13.039	3.356	27	0.007*
	Pre vs 1M	8.903	2.419	2.73	15.077	3.68	27	0.003*
	Post vs 1M	1.497	1.671	−2.767	5.762	0.896	27	1.000
PSQI	Pre vs Post	0.286	0.457	−1.452	0.88	0.626	27	1.000
	Pre vs 1M	0.786	0.422	−0.292	1.863	1.863	27	0.221
	Post vs 1M	1.071	0.398	0.055	2.088	2.691	27	0.036*
POMS	Pre vs Post	6.071	4.858	6.329	18.472	1.25	27	.666
	Pre vs 1M	13.464	3.495	4.543	22.385	3.852	27	.002*
	Post vs 1M	7.393	4.893	5.096	19.882	1.511	27	.427

*p indicates statistical significance.

For the simulator sickness questionnaire (SSQ), the total score was calculated using the following formula: total score = nausea score + oculomotor score + disorientation × 3.74) [16]. The nausea, oculomotor, disorientation, and total scores of our system were 75, 70.57, and 106.47, respectively (Table 4). The total score was 962.68 points, as calculated using the aforementioned formula.

**Table 4:** Analysis of nausea, oculomotor, disorientation, and total score derived from the SSQ.

SSQ			
Nausea (SD)	Oculomotor (SD)	Disorientation (SD)	Total Score (SD)
75 (15.58)	70.57 (20.57)	106.47 (21.47)	962.68 (194.84)

SSQ, simulator sickness questionnaire; SD, standard deviation.

### Sensor-level power spectrum analysis

Significant increases in power spectrum density (PSD) in the gamma band (30–55 Hz) were observed in the right parietal-occipital and left central regions of patients with tinnitus following VR intervention. After the intervention, there was a notable enhancement in the relative power of the gamma band in these regions, specifically at the right parietal-occipital (p = 0.030) and left central (p = 0.042) electrodes. Additionally, a significant improvement in alpha asymmetry was detected at the AF3 and AF4 electrodes (p = 0.025) immediately after the intervention compared to the pre-intervention measurements (Table 5). No significant differences were observed in the measurements between the pre-intervention phase and 1-month post-intervention.

**Table 5:** Mean values and standard deviations of significant features in power spectrum density analysis and associated source activities.

		Pre-intervention	Post-intervention	p-value
Sensor level	Alpha asymmetry AF3–AF4	−2.03 ± 6.24	1.04 ± 5.19	0.025
	Gamma right PO	0.27 ± 0.17	0.31 ± 0.18	0.030
	Gamma left central	0.34 ± 0.27	0.47 ± 0.42	0.042
Source level	Low beta right MFG	55.74 ± 39.44	69.03 ± 57.35	0.045
	Gamma left IPL	2.36 ± 1.87	3.44 ± 3.22	0.049
	Gamma right MFG	5.43 ± 4.47	7.37 ± 7.27	0.037
	Gamma left precuneus	2.21 ± 1.75	3.11 ± 2.73	0.043

Left central includes C3, T3, FC1, FC5, CP1, and CP5 electrodes.

Right PO includes T6, P4, and O2 electrodes.

PO, parietal occipital; MFG: middle frontal gyrus; IPL, inferior parietal lobule.

### Source-level power spectrum analysis

**Fig 6:**
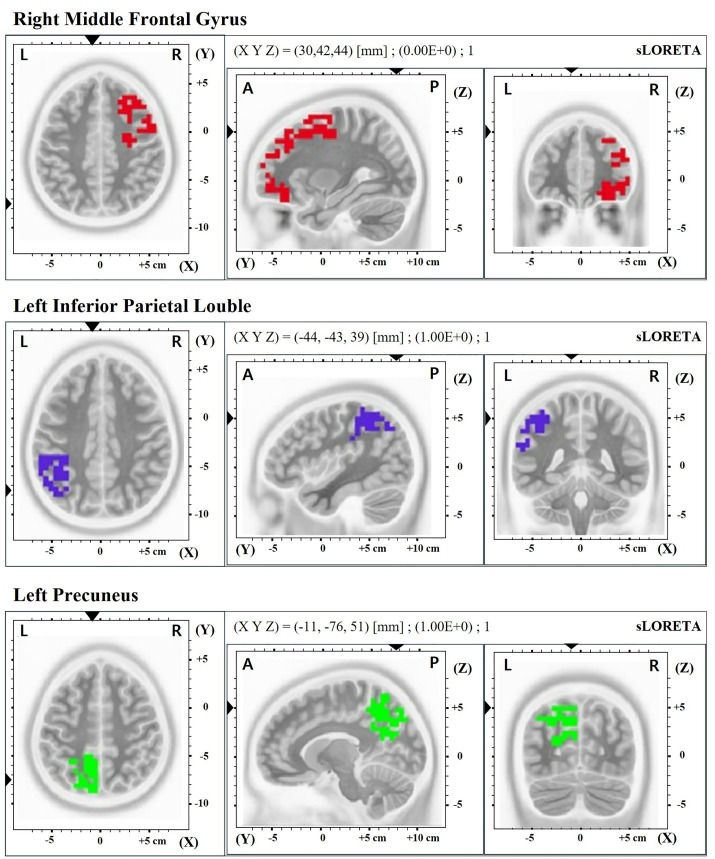
Brain regions with significant differences in source level features before and after the VR intervention. VR, virtual reality.

Following the intervention, patients with tinnitus demonstrated a significant increase in gamma-band source activity across multiple brain regions, notably in the right middle frontal gyrus (p = 0.037), left inferior parietal lobule (p = 0.049), and left precuneus (adjusted p = 0.043). Additionally, a marked activation in the low-beta band (12–18 Hz) was observed specifically in the right middle frontal gyrus when comparing data from the post-intervention phase to those from the pre-intervention phase (p = 0.045; Table 3 and Fig 6). However, no significant differences were observed in the measurements when comparing the pre-intervention phase with 1-month post-intervention.

## Discussion

This study assessed the effectiveness of a VR-based psychological intervention system as part of behavioral cognitive therapy for patients with tinnitus, utilizing a VR system with high visual, auditory, and tactile realism. We hypothesized that this method could aid in the recovery of patients with severe tinnitus. Analysis of the questionnaires indicated that VR-based intervention reduced tinnitus and its related symptoms. The THQ, which measures the severity of tinnitus-related distress and is useful for predicting psychological distress, varied between individuals, and scores improved in 21 of 28 patients.

In our questionnaire results, the THQ scores significantly improved immediately after VR therapy compared with those at baseline before VR therapy; however, no significant differences were observed in the other questionnaire results immediately after treatment. Nevertheless, 1-month after treatment, THQ scores tended to decrease further compared to the baseline scores before VR therapy, and both the PSQI scores, indicating improved sleep quality, and the POMS scores significantly decreased. This indicates that the effect of VR therapy manifests as long-term score changes on survey indicators beyond immediate post-treatment effects. Similar results showing long-term improvement in survey scores compared to that immediately after treatment have been found in previous studies on VR tinnitus therapy. In this study, despite no significant changes immediately after treatment and at 1-month compared to before VR-tinnitus therapy, all scores for the STSS, THQ, HADS, and visual analog scale (VAS) showed significant improvement 3 months after treatment compared to that before treatment [6].

Based on the SSQ analysis, our VR system exhibited a relatively low level of motion sickness, with an SSQ score of 962.68. The low SSQ score suggests that the VR system did not induce motion sickness.

Previous studies have established that individuals with tinnitus exhibit greater PSD in the gamma band when subjected to acoustic residual inhibition (ARI) than that in healthy controls [17–19]. ARI refers to a phenomenon where the perceived intensity of tinnitus temporarily decreases after exposure to a specific auditory stimulus. This reduction in tinnitus loudness is attributed to inhibitory neural processes triggered by the stimulus, leading to a transient suppression of tinnitus perception once the stimulus ends [19]. In this study, individualized sound exposure, particularly at frequencies matching the patient’s tinnitus pitch, was employed to induce the desired inhibitory effect. Transient and partial normalization of deafferentation in the auditory thalamus leads to the occurrence of ARI. This process suggests that the temporary re-establishment of neural inputs or connectivity in the deafferented auditory thalamus contributes to the reduction of tinnitus symptoms [17]. King et al. observed a higher degree of PSD, specifically in the gamma band, during ARI than during the control period [17]. Their findings revealed a noteworthy association between gamma activity and residual inhibition, underscoring the potentially advantageous effects on neurological reactions in individuals with tinnitus. This implies that gamma activity may have a significant impact on modulating auditory symptoms linked to tinnitus; therefore, its therapeutic implications warrant further research.

In the present study, tinnitus-like noise generated via the VR environment consequently induced ARI and significantly enhanced gamma PSD, specifically in the right parieto-occipital and left central regions of the study population. In a magnetoencephalography (MEG) study, the parieto-occipital region has been reported as an important component of the cortical networks of chronic tinnitus [20]. A resting-state EEG study identified a negative correlation between gamma band activity in the parieto-occipital region and VAS scores, indicating that higher gamma activity corresponds to lower perceived tinnitus severity [21]. Another resting-state EEG study revealed an increase in brain activity in the central region associated with tinnitus [22].

Additionally, a previous resting-state EEG study identified significantly higher activity in the gamma band of the inferior parietal lobule (IPL). A recent MEG study proposed that the inferior parietal regions, which are associated with auditory memory and awareness, function as the central regions in the tinnitus network [23,24]. Furthermore, gamma functional connectivity in the left middle frontal gyrus (MFG) and left precuneus was significantly reduced in the tinnitus group compared to that in the control group, according to a recent functional magnetic resonance imaging study [25]. The precuneus is frequently regarded as a critical node of the default mode network (DMN), which comprises a distinct cluster of brain regions that become active when individuals engage in internally focused activities [26]. Lee et al. suggested that tinnitus perception may be linked to active connections with the DMN, which processes prominent yet unrelated auditory information (i.e., tinnitus) as normal. This leads to persistent tinnitus perception, highlighting an abnormality in the precuneus of the tinnitus group [27]. With regard to the middle frontal gyrus, decreased regional homogeneity values in the right MFG were observed in patients with tinnitus compared to those in the control group [28]. According to Aldhafeeri et al., the cortical thickness of the bilateral MFG was structurally altered in patients with severe tinnitus [29].

As suggested in previous studies, these regions may play a pivotal role in elucidating the pathophysiology of tinnitus. Moreover, our findings indicate that alterations in neurological responses to tinnitus elicited by the VR intervention may represent a viable therapeutic avenue for modulating tinnitus symptoms. This underscores the potential utility of VR as a novel intervention strategy for the treatment of tinnitus and merits further investigation.

However, this study has certain limitations. First, the small sample size and short treatment duration did not result in significant functional differences through CBT. Although we believe that the method may have an effect on tinnitus-related distress by increasing a sense of “control,” future studies should measure and examine the psychological processes underlying the observed effects. Several participants performed poorly in the study owing to the lack of popularity of VR systems among the general public. To improve VR-based therapy, providing more scenes and increasing patient exposure to virtual reality is recommended. Additionally, a control group was not included because of the inability to administer interventions to patients. When designing the control group, it was not possible to include individuals without tinnitus. However, future studies should examine the effect of the intervention on patients with chronic tinnitus compared to those with other functional hearing symptoms, such as migraine, stridor, and hyperacusis, which are not medically explained. Additionally, it is important to note that statistically significant changes may not always translate into clinically meaningful changes. Therefore, future studies should focus on identifying the factors that predict clinically meaningful changes in the patient population. Furthermore, the EEG analysis did not apply multiple comparison corrections. The analysis focused solely on the treatment effect, without considering demographic factors as covariates. A more comprehensive and conservative approach, including these factors and corrections, should be explored in future studies. (Finally, each patient had different hearing levels, which may have influenced the results because of the disparity in tinnitus mechanisms between patients with normal hearing and those with hearing loss. Despite these limitations, this study is expected to be a steppingstone towards improved methods for VR-based treatment of patients with tinnitus.

In conclusion, chronic subjective tinnitus can have a range of negative effects on patients’ lives, and effective treatment options are currently limited. CBT has gained attention as a potential treatment, and our research has expanded upon previous studies by incorporating graphics and other enhancements. However, this approach requires considerable patient attention and compliance. Our research team aimed to evaluate the potential of VR intervention systems to provide users with realistic experiences as part of CBT. We observed relief of tinnitus symptoms and changes in EEG patterns related to the tinnitus-generating system. However, owing to time and material limitations, the intervention did not produce significant changes. Overcoming these limitations will enable the discovery of new methods of treating patients with tinnitus.

## Supporting information

S1 Table Presents the statistical summary of measured variables across three time points. (DOCX)

S2 Table Statistical comparisons across three time points. (DOCX)

S1 Dataset De-identified individual-level questionnaire response data underlying all reported means and statistical analyses. (XLSX)
