# Supplementary material for: Multi-modal virtual reality system for tinnitus treatment methods and validation
Source: PLoS One. 2025 Sep 8;20(9):e0330843. doi: 10.1371/journal.pone.0330843 (PMC12416703; doi:10.1371/journal.pone.0330843)
Supplement: S1 Table — (DOCX) [file pone.0330843.s001.docx]

**Table S1 Presents the statistical summary of measured variables across three time points: before treatment (Pre), immediately after treatment (Pos), and one month after treatment (1Month). The values for Mean (M) and Standard Deviation (SD) are rounded to four decimal places for clarity and consistency.**

| **Measure** | **Group** | **Mean(M)** | **N** | **sd** |
| --- | --- | --- | --- | --- |
| THQ | Pre | 47.347 | 28 | 19.641 |
|  | Post | 39.940 | 28 | 17.953 |
|  | 1Month | 38.443 | 28 | 18.438 |
| PSQI | Pre | 9.18 | 28 | 4.53 |
|  | Post | 9.46 | 28 | 3.626 |
|  | 1Month | 8.39 | 28 | 3.624 |
| WQOL | Pre | 78.571 | 28 | 12.509 |
|  | Post | 76.464 | 28 | 13.290 |
|  | 1Month | 77.500 | 28 | 13.423 |
| POMS | Pre | 76.107 | 28 | 21.588 |
|  | Post | 70.036 | 28 | 27.197 |
|  | 1Month | 62.643 | 28 | 27.228 |
| HADS  - Anxiety | Pre | 6.929 | 28 | 2.508 |
|  | Post | 7.036 | 28 | 2.822 |
|  | 1Month | 6.71 | 28 | 2.98 |
| HADS - Depression | Pre | 7.036 | 28 | 2.715 |
|  | Post | 7.393 | 28 | 2.544 |
|  | 1Month | 7.143 | 28 | 3.205 |
| VNS  - Duration | Pre | 77.14 | 28 | 22.584 |
|  | Post | 76.07 | 28 | 24.242 |
|  | 1Month | 69.179 | 28 | 31.121 |
