# Supplementary material for: Multi-modal virtual reality system for tinnitus treatment methods and validation
Source: PLoS One. 2025 Sep 8;20(9):e0330843. doi: 10.1371/journal.pone.0330843 (PMC12416703; doi:10.1371/journal.pone.0330843)
Supplement: S2 Table — (DOCX) [file pone.0330843.s002.docx]

**Table S2.** **Statistical comparisons across three time points: Pre (before treatment), Pos (immediately after treatment), and 1Month (one month after treatment). Significant p-values (p < 0.05) are marked with an asterisk (*).**

| **Measure** | **Comparison** | **Pearson**  **Correlation Coefficient** | **t-statistic** | **Degrees of freedom** | **p-Value** |
| --- | --- | --- | --- | --- | --- |
| THQ | Pre vs Post | 0.811 | 3.356 | 27 | 0.002* |
|  | Pre vs 1M | 0.776 | 3.681 | 27 | <0.001* |
|  | Post vs 1M | 0.882 | 0.896 | 27 | 0.378 |
| PSQI | Pre vs Post | 0.847 | -0.626 | 27 | 0.537 |
|  | Pre vs 1M | 0.873 | 1.861 | 27 | 0.074 |
|  | Post vs 1M | 0.831 | 2.691 | 27 | 0.012* |
| WQOL | Pre vs Post | 0.889 | 1.821 | 27 | 0.080 |
|  | Pre vs 1M | 0.905 | 0.990 | 27 | 0.331 |
|  | Post vs 1M | 0.932 | -1.113 | 27 | 0.276 |
| POMS | Pre vs Post | 0.464 | 1.250 | 27 | 0.222 |
|  | Pre vs 1M | 0.736 | 3.852 | 27 | <0.001* |
|  | Post vs 1M | 0.547 | 1.511 | 27 | 0.142 |
| HADS - Anxiety | Pre vs Post | 0.665 | -0.258 | 27 | 0.799 |
|  | Pre vs 1M | 0.765 | 0.587 | 27 | 0.562 |
|  | Post vs 1M | 0.807 | 0.942 | 27 | 0.355 |
| HADS - Depression | Pre vs Post | 0.770 | -1.056 | 27 | 0.300 |
|  | Pre vs 1M | 0.795 | -0.291 | 27 | 0.773 |
|  | Post vs 1M | 0.702 | 0.574 | 27 | 0.571 |
| VNS  - Duration | Pre vs Post | 0.757 | 0.346 | 27 | 0.732 |
|  | Pre vs 1M | 0.790 | 2.197 | 27 | 0.037* |
|  | Post vs 1M | 0.699 | 1.629 | 27 | 0.115 |
